# The potential impact of clinical factors on blood-based biomarkers for Alzheimer’s disease

**DOI:** 10.1186/s40035-023-00371-z

**Published:** 2023-08-18

**Authors:** Fengfeng Pan, Yan Lu, Qi Huang, Fang Xie, Jingye Yang, Qihao Guo

**Affiliations:** 1 Department of Gerontology, Shanghai Sixth People’s Hospital Affiliated to Shanghai Jiao Tong University School of Medicine, Shanghai, 200233 China; 2 PET Center, Huashan Hospital, Fudan University, Shanghai, 200233 China

With the development of testing technologies, blood-based biomarkers for Alzheimer’s disease (AD) such as amyloid-β (Aβ), phosphorylated tau (P-tau), and neurofilament light (NfL) have shown potential value in predicting AD pathology and disease progression [1]. To better interpret the detection results, it is essential to understand what factors would affect the concentrations of these biomarkers. While standardized testing methods may reduce the influence of pre-analytical factors, demographic factors and clinical comorbidities may still affect blood biomarker levels [2, 3]. Previous studies have shown inconsistent results in this regard. For example, the apolipoprotein E (*APOE*) ε4 genotype was associated with a lower Aβ42/Aβ40 ratio in a cohort with different cognitive status but not in a population with or without AD [2, 4]. In a cohort where most participants had no cognitive impairment, males had higher plasma P-tau levels than females, whereas this was not the case in another group composed mostly of cognitively impaired participants [3, 5]. Given that the blood-based biomarkers are inevitably associated with AD pathology and cognitive decline, the inconsistent results may be attributed to the different severities of AD pathology and cognitive dysfunction in different research cohorts. Here, we performed a retrospective cross-sectional single-center study to investigate the effects of common demographic factors and clinical comorbidities on the plasma levels of Aβ42/Aβ40, T-tau, P-tau181, and NfL in a cohort with different cognitive status. Clinical comorbidities were self-reported or extracted from patient records. Effects were adjusted for age and brain Aβ burden determined by ^18^F-florbetapir (AV45) PET standard uptake value ratios (SUVRs). Detailed methods are provided in Additional file 1. A total of 685 participants were recruited, including 401 cognitively normal (CN) individuals and 284 individuals with cognitive impairment (CI). Individuals with CI were defined as having performance in standardized neuropsychological tests more than one standard deviation below the age-corrected normative mean. Demographic and clinical characteristics are shown in Table S1. The associations of plasma biomarkers with age and AV45 SUVRs are shown in Figures S1, Table S2, and Figure S2. All these tables and figures can be found in Additional file 2. The effects of demographic factors and clinical comorbidities on plasma biomarkers are displayed in Fig. 1.

**Fig. 1:**
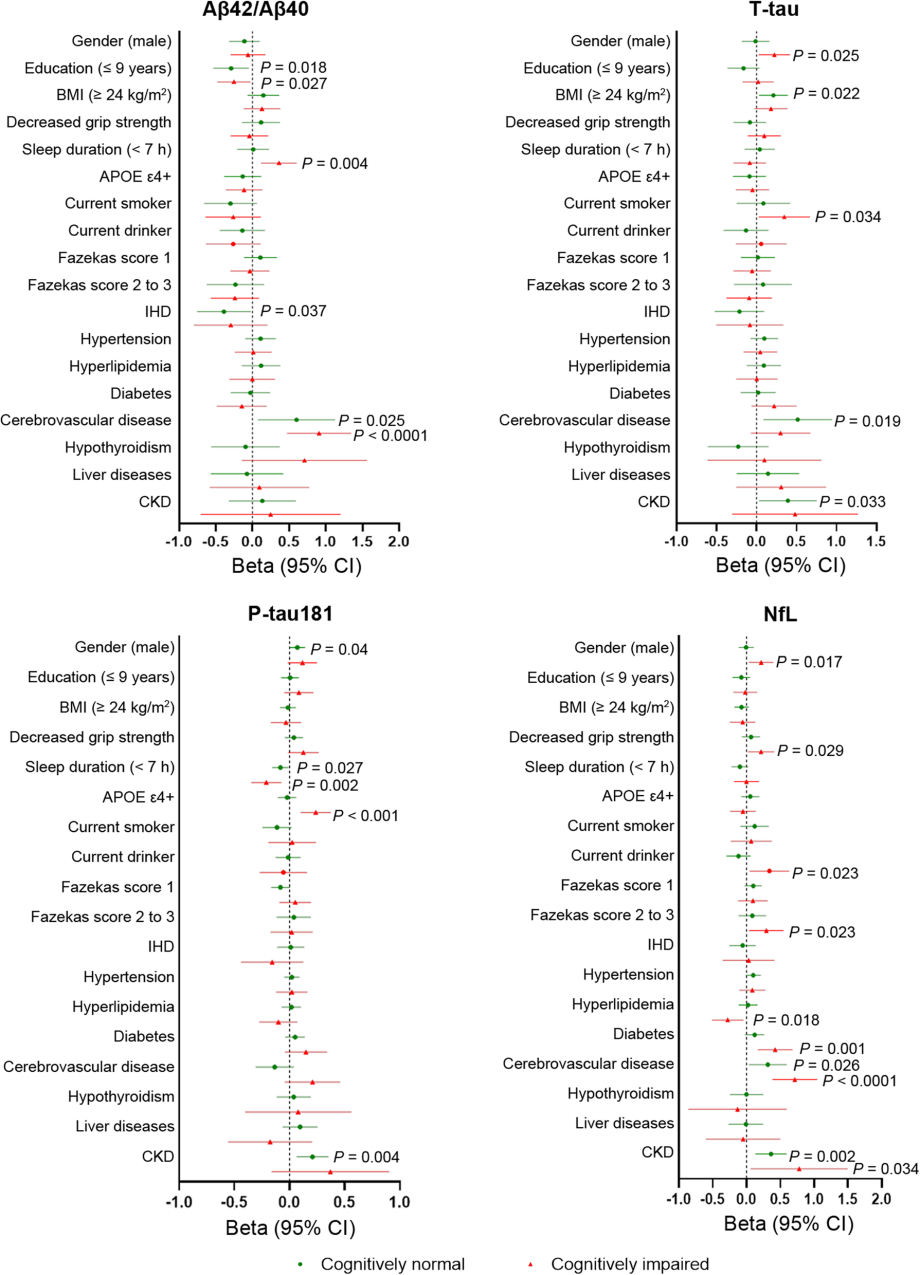
Forest plots of linear model results adjusted for age and AV-45 PET SUVR. Beta coefficient represents the parameter estimate for the linear regression model. Green and red lines represent the 95% confidence interval in cognitively normal and cognitively impaired individuals, respectively. BMI, body mass index; APOE, apolipoprotein E; IHD, Ischemic heart disease; CKD, Chronic kidney disease

In the CN group, the plasma Aβ42/Aβ40 ratio had a negative correlation with age (*r* =  − 0.244, *P* < 0.0001) and Aβ-PET SUVRs (*r* =  − 0.1417, *P* = 0.005). Similarly, in CI subjects, there was a negative correlation of the ratio with age (*r* =  − 0.161, *P* = 0.006) and Aβ-PET SUVRs (*r* =  − 0.1705, *P* = 0.004). In the CN group, cerebrovascular disease was associated with a higher plasma Aβ42/Aβ40 ratio (Beta = 0.600, *P* = 0.025). Lower education and ischemic heart disease (IHD) showed a correlation with a lower Aβ42/Aβ40 ratio (Beta =  − 0.291, *P* = 0.018 and Beta =  − 0.389, *P* = 0.037, respectively). In the CI participants, similar effects were observed for lower education and cerebrovascular disease (Beta =  − 0.253, *P* = 0.027 and Beta = 0.909, *P* < 0.001, respectively), while a shorter nighttime sleep duration was associated with a higher Aβ42/Aβ40 ratio (Beta = 0.361, *P* = 0.004).

Plasma P-tau181 was positively correlated with age and Aβ-PET SUVR (*r* = 0.212, *P* < 0.0001 and *r* = 0.179, *P* = 0.0004, respectively) in CN participants and positively correlated with Aβ-PET SUVR (*r* = 0.356, *P* < 0.0001) but not age in CI participants. In the CN group, males and individuals with chronic kidney disease (CKD) were more likely to have a higher level of plasma P-tau181 (Beta = 0.071, *P* = 0.040 and Beta = 0.209, *P* = 0.004, respectively). A shorter nighttime sleep duration was associated with a lower level of P-tau181 (Beta =  − 0.083, *P* = 0.027). In the CI participants, a similar effect was observed for the shorter nighttime sleep duration (Beta =  − 0.212, *P* = 0.002), while *APOE* ε4 carriers were more likely to have a higher level of P-tau181 (Beta = 0.237,* P* < 0.001).

Plasma NfL had significant positive correlations with age in both CN and CI participants (*r* = 0.461, *P* < 0.0001 and* r* = 0.257, *P* < 0.0001, respectively). No significant correlation was found between plasma T-tau and age. Plasma NfL had no significant correlation with Aβ-PET SUVR in either group, while plasma T-tau positively correlated with Aβ-PET SUVR in the CN participants (*r* = 0.125, *P* = 0.014). In the CN participants, cerebrovascular disease and CKD were associated with higher plasma T-tau (Beta = 0.514, *P* = 0.019 and Beta = 0.390,* P* = 0.033, respectively) and NFL (Beta = 0.315, *P* = 0.026 and Beta = 0.361, *P* = 0.022, respectively), and a higher body mass index (BMI) was also associated with a higher plasma T-tau level (Beta = 0.21, *P* = 0.022). In the CI participants, males had higher plasma T-tau and NfL levels than females (Beta = 0.222, *P* = 0.025 and Beta = 0.216, *P* = 0.017). Smoking consumption was associated with higher plasma T-tau (Beta = 0.346, *P* = 0.034). Plasma NfL level was more likely to be higher in individuals with decreased handgrip strength (Beta = 0.215, *P* = 0.029), alcohol consumption (Beta = 0.339, *P* = 0.023), moderate-to-severe white matter hyperintensities (Fazekas 2–3) (Beta = 0.293, *P* = 0.023), diabetes (Beta = 0.424, *P* = 0.001), cerebrovascular disease (Beta = 0.714, *P* < 0.001), and CKD (Beta = 0.778, *P* = 0.034), but lower in individuals with hyperlipidemia (Beta = −0.278, *P* = 0.0118).

The negative correlations of plasma Aβ42/Aβ40 ratio with age and Aβ-PET SUVR in our study indicate that a lower plasma Aβ42/Aβ40 ratio could be not only caused by the pathological change in AD but also associated with advancing age. We found that plasma P-tau181 was positively correlated with age only in the CN subjects, while there was a stronger correlation between plasma P-tau181 and Aβ-PET SUVR in CI subjects compared to CN subjects. In contrast, plasma NfL had no significant association with Aβ-PET SUVR but positively correlated with age in both cognitive status. These associations indicate that plasma P-tau181 is a more AD-specific biomarker, while NfL is a more age-related neurodegeneration biomarker. In our research, males had higher plasma T-tau, NfL, and P-tau181 levels than females. In addition, individuals with lower education had a lower plasma Aβ42/Aβ40 ratio. These all suggest population-specific reference ranges for these plasma biomarkers. Our study found that the *APOE* ε4 genotype had no significant effect on plasma Aβ42/Aβ40 ratio after adjusting for the brain Aβ burden. However, in the CI group, the *APOE* ε4 carriers had a significantly higher level of P-tau181 than non-carriers, indicating a potential Aβ-independent mechanism for the *APOE* ε4 genotype effect in AD [6].

Previous reports showed that shorter nighttime sleep duration led to a higher P-tau level in the cerebrospinal fluid [7]. Unexpectedly, here we found a lower level of plasma P-tau181 in our subjects with shorter nighttime sleep duration. In addition, a higher plasma Aβ42/Aβ40 ratio was observed in our CI participants with shorter nighttime sleep duration. These suggest that in individuals with sleep disorders, the predictive abilities of plasma Aβ42/Aβ40 and P-tau181 for AD may be reduced.

In this study, a lower plasma Aβ42/Aβ40 ratio was observed in the CN participants with IHD. This might be due to the effects of peripheral blood Aβ on the pathophysiological changes of IHD [8]. By contrast, the plasma Aβ42/Aβ40 ratio and the plasma NfL were significantly higher in the individuals with cerebrovascular disease. Therefore, the predictive accuracy of these plasma biomarkers for brain Aβ deposition and AD progression may be affected in a population with cardiovascular or cerebrovascular diseases. A previous study has noted elevated levels of processed APP products in connection with lowered thyroid hormone [9]. In addition, impaired liver or renal function may influence the metabolism of blood Aβ peptides and lead to higher levels of Aβ42 and Aβ40 [2, 10]. However, our research did not establish any correlation between plasma Aβ42/Aβ40 and hypothyroidism, chronic liver disease, or CKD. This could be due to both plasma Aβ42 and Aβ40 experiencing the same alteration in the individuals with these comorbidities. Nevertheless, our study revealed that the plasma levels of T-tau, P-tau181, and NfL were more likely to rise with the clinical comorbidity of CKD. Therefore, glomerular filtration rate should be taken into consideration during the use and the interpretation of these plasma biomarkers.

In conclusion, the plasma levels of AD biomarkers are affected by multiple demographic factors and clinical comorbidities. Considerations of the relevant factors are essential for the clinical interpretability and applicability of the biomarkers.

### Supplementary Information

**Additional file 1: Methods.**

**Additional file 2: Table S1.** Clinical characteristics and plasma biomarkers of the study participants. **Table S2.** Comparisons of plasma biomarkers across age groups in different cognitive status. **Fig. S1.** Correlations between plasma biomarkers and age. **Fig. S2.** Correlations between plasma biomarkers and brain Aβ burden.

## Data Availability

The datasets used and/or analyzed during the current study are available from the corresponding authors on reasonable request.

## Abbreviations

AD: Alzheimer’s disease
Aβ: Amyloid beta
APOE: Apolipoprotein E
BMI: Body mass index
CKD: Chronic kidney disease
IHD: Ischemic heart disease
NfL: Neurofilament light
P-tau: Phosphorylated tau
SUVR: Standard uptake value ratios
