# Supplementary material for: The potential impact of clinical factors on blood-based biomarkers for Alzheimer’s disease
Source: Transl Neurodegener. 2023 Aug 18;12:39. doi: 10.1186/s40035-023-00371-z (PMC10436434; doi:10.1186/s40035-023-00371-z)
Supplement: Supplementary file 1 — Additional file 1: Methods. [file 40035_2023_371_MOESM1_ESM.docx]

**Methods**

**Study participants**

Mandarin-speaking participants were recruited from the Shanghai cognitive-friendly community and outpatients in the geriatric department of Shanghai Sixth People's Hospital from January 2019 to June 2021. The inclusion criteria for this study were as follows: aged 40-85 years, educated for more than one year, had the ability to finish a battery of neuropsychological tests, underwent cranial MRI and 18F-florbetapir PET scans within three months after cognitive assessments and blood sampling, be able to give informed consent and complete the examinations of blood-based biomarkers. Written informed consent was obtained from all the participants or their caregivers. The ethics committee of Shanghai Sixth People’s Hospital Affiliated to Shanghai Jiao Tong University School of Medicine reviewed and approved this study.

**Demographic factors and clinical comorbidities**

Demographic factors which may have effects on blood biomarkers were obtained at the time of cognitive assessments, including sex, age, body mass index (BMI), years of education, smoking and alcohol consumption, and nighttime sleep duration. Grip strength was evaluated using a grip strength meter, and decreased grip strength was defined as ≤28 kg/m^2^ for men and ≤18 kg/m^2^ for women. Apolipoprotein E (APOE) genotype was determined by polymerase chain reaction (PCR) followed by direct sequencing and categorized into APOE ε4 carriers and noncarriers. White matter hyperintensities (WMH) burden was assessed by Siemens Prisma 3.0T magnetic resonance imaging scan. In brief, WMH was defined as clearly hyperintense areas relative to surrounding white matter on both FLAIR and T 2-weighted images. The WMH burden was graded according to Fazekas visual score (0, 1, 2, 3)(1). Clinical comorbidities were based on subject self-report or extracted from patient records, including hypertension, diabetes, hyperlipidemia, ischemic heart disease (IHD), cerebrovascular disease, hypothyroidism, chronic liver disease, chronic kidney disease (CKD). In more detail, IHD was determined by invasive coronary angiography or noninvasive cardiac imaging modalities. Cerebrovascular disease was defined as a history of ischemic or hemorrhagic stroke determined by a CT or MRI scan of the brain. Chronic liver disease was defined if the participants had a history of chronic hepatitis C, chronic hepatitis B, alcoholic liver disease or metabolic-associated fatty liver disease. Chronic kidney disease was defined if the participants had a prior history of histological changes in kidney biopsy, previous kidney transplantation, or a history of renal injury for more than three months, including glomerular filtration rate (GFR) lower than 60 ml/min, albuminuria, and hematuria.

**Cognitive assessment and classification**

The global cognitive function was assessed by the Chinese version of Mini-Mental State Examination (MMSE)(2), Montreal Cognitive Assessment-Basic (MoCA-BC)(3), and Addenbrooke’s Cognitive Examination III (ACE-III-CV)(4). A battery of standardized neuropsychological tests was used to assess different cognitive domains, including Auditory Verbal Learning Test (AVLT) 20-minute delayed free recall and AVLT recognition for memory(5), Boston Naming Test (BNT), and Animal Verbal Fluency Test (AFT) for language(6, 7), Shape Trail Test Part A and B (STT-A, STT-B) for executive function(8), Judgement of Line Orientation(JLO) for visuospatial ability(9), and Symbol Digit Modalities Test (SDMT) and Digit Span Test (DST) for attention(10). Impaired scores of these neuropsychological tests were defined as >1 standard deviation (SD) below the age-corrected normative mean. The functional status of daily life was assessed by Activities of Daily Living (ADL) and Functional Assessment Questionnaire (FAQ)(11). The Hamilton Depression Scale (HAMD) and Hamilton Anxiety Scale (HAMA) were used to evaluating mood and anxiety symptoms, respectively(12, 13). Participants were classified into cognitively normal (CN) and cognitively impaired (CI) groups according to their performances on the standardized neuropsychological tests. In brief, participants with or without cognitive complaints but normally performed on the standardized neuropsychological tests were defined as CN. Those with self-reported or informants identified cognitive decline and had objective cognitive impairment verified via standardized neuropsychological tests were defined as CI.

**Brain Amyloid Burden**

18F-florbetapir (AV45) PET imaging was acquired with a PET/CT system (Biograph mCT Flow PET/CT, Siemens, Erlangen, Germany) to assess the brain amyloid burden. PET scans were performed 50min after the intravenous injection of 7.4 MBq/kg (0.2mCi/kg) 18F-florbetapir and lasted for 20min. PET images were reconstructed by filtered back projection algorithm with corrections for decay, normalization, dead time, photon attenuation, scatter, and random coincidences. The brain amyloid burden was determined by the standard uptake value ratio (SUVR) using cerebellar crus as a reference, which is widely used as a proxy for the quantitative analysis of amyloid PET. A global AV45 PET SUVR value was estimated for each participant by weighted averaging of the cortical regions of interest (ROIs), including frontal gyrus, lateral parietal gyrus, lateral temporal gyrus, medial temporal gyrus, posterior cingulate gyrus, and precuneus(14).

**Measurements of plasma biomarkers**

Plasma samples were collected from EDTA blood, followed by centrifuged, aliquoted, and stored at -80 ºC. All the plasma samples went through one freeze-thaw cycle before the examination. Plasma biomarkers were measured on the Quanterix Simoa HD-1 platform(15). Neurology 3-Plex A Assay Kit (Lot 502838) was used to measure Aβ42, Aβ40 and T-tau, P-Tau 181 Assay Kit V2 (Lot 502923) was used to measure P-tau, and NF-light Assay Kit (Lot 202700) was used to measure NfL. In brief, plasma samples were diluted 1:4 according to the minimum required dilution (MRD). Plasma samples, reagents including magnetic beads encapsulated with capture antibody, biotinylated detection antibody, streptavidin-β galactosidase, and β-D-galactopyranoside were successively loaded on the plate to generate detectable signals. Concentrations of plasma biomarkers (pg/mL) were determined by the four-parameter curve fit algorithm using Simoa HD-1 Analyzer software. Biomarker measurements were performed by laboratory technicians blinded to the clinical data.

**Statistical analyses**

For between-group comparisons, categorical variables were expressed as numbers (percentages) and analyzed by the Chi-squared test. Continuous variables were expressed as median (interquartile range) and analyzed by the Mann-Whitney U test. Spearman correlation analysis was used to evaluate the relationships between age, Aβ-PET SUVR, and plasma Aβ42, Aβ40, Aβ42/Aβ40 ratio, T-tau, P-tau181, and NfL in different cognitive statuses. General linear models were used to determine the effects of demographic factors and clinical comorbidities on the levels of plasma biomarkers in the participants with CN and CI, respectively. Given that age and Aβ-PET SUVR had significant correlations with most plasma biomarkers and were demonstrated to be associated with multiple clinical characteristics and clinical comorbidities, these two factors were adjusted for in the models. Response coefficients (betas) were estimated for each factor, including male sex, lower education (≤9 years), higher BMI (≥24kg/m2), decreased handgrip strength (≤28 kg/m^2^ for men and ≤18 kg/m^2^ for women), shorter nighttime sleep duration (<7 hours), smoking consumption, alcohol consumption, APOE ε4 carrier, higher WMH burden (Fazekas score 1 and 2-3), hypertension, diabetes, hyperlipidemia, IHD, cerebrovascular disease, hypothyroidism, chronic liver disease, and CKD. All plasma biomarkers were z-scored within participants to compare coefficients. A two-sided P value <0.05 was considered statistically significant. All data analyses were performed with IBM SPSS Statistics 23.0. A graphics package (GraphPad Prism, version 8.0) was used to create figures.

**REFERENCES**

1. Pantoni L, Basile AM, Pracucci G, Asplund K, Bogousslavsky J, Chabriat H, et al. Impact of age-related cerebral white matter changes on the transition to disability -- the LADIS study: rationale, design and methodology. Neuroepidemiology. 2005;24(1-2):51-62.

2. Katzman R, Zhang MY, Ouang Ya Q, Wang ZY, Liu WT, Yu E, et al. A Chinese version of the Mini-Mental State Examination; impact of illiteracy in a Shanghai dementia survey. J Clin Epidemiol. 1988;41(10):971-8.

3. Chen KL, Xu Y, Chu AQ, Ding D, Liang XN, Nasreddine ZS, et al. Validation of the Chinese Version of Montreal Cognitive Assessment Basic for Screening Mild Cognitive Impairment. J Am Geriatr Soc. 2016;64(12):e285-e90.

4. Pan FF, Wang Y, Huang L, Huang Y, Guo QH. Validation of the Chinese version of Addenbrooke's cognitive examination III for detecting mild cognitive impairment. Aging Ment Health. 2022;26(2):384-91.

5. Zhao Q, Guo Q, Liang X, Chen M, Zhou Y, Ding D, et al. Auditory Verbal Learning Test is Superior to Rey-Osterrieth Complex Figure Memory for Predicting Mild Cognitive Impairment to Alzheimer's Disease. Curr Alzheimer Res. 2015;12(6):520-6.

6. Kaplan E, Goodglass H, Weintrab S. The Boston Naming Test. Philadelphia: Lea & Febiger. 1983.

7. Zhao Q, Guo Q, Hong Z. Clustering and switching during a semantic verbal fluency test contribute to differential diagnosis of cognitive impairment. Neurosci Bull. 2013;29(1):75-82.

8. Zhao Q, Guo Q, Li F, Zhou Y, Wang B, Hong Z. The Shape Trail Test: application of a new variant of the Trail making test. PLoS One. 2013;8(2):e57333.

9. Qualls CE, Bliwise NG, Stringer AY. Short forms of the Benton Judgment of Line Orientation Test: development and psychometric properties. Arch Clin Neuropsychol. 2000;15(2):159-63.

10. Wechsler D. Wechsler Adult Intelligence Scale—Fourth edition: Technical and interpretive manual. 2008.

11. Pfeffer RI, Kurosaki TT, Harrah CH, Jr., Chance JM, Filos S. Measurement of functional activities in older adults in the community. J Gerontol. 1982;37(3):323-9.

12. Zimmerman M, Martinez JH, Young D, Chelminski I, Dalrymple K. Severity classification on the Hamilton Depression Rating Scale. J Affect Disord. 2013;150(2):384-8.

13. Maier W, Buller R, Philipp M, Heuser I. The Hamilton Anxiety Scale: reliability, validity and sensitivity to change in anxiety and depressive disorders. J Affect Disord. 1988;14(1):61-8.

14. Akamatsu G, Ikari Y, Ohnishi A, Nishida H, Aita K, Sasaki M, et al. Automated PET-only quantification of amyloid deposition with adaptive template and empirically pre-defined ROI. Phys Med Biol. 2016;61(15):5768-80.

15. Wilson DH, Rissin DM, Kan CW, Fournier DR, Piech T, Campbell TG, et al. The Simoa HD-1 Analyzer: A Novel Fully Automated Digital Immunoassay Analyzer with Single-Molecule Sensitivity and Multiplexing. J Lab Autom. 2016;21(4):533-47.
