# Supplementary material for: The potential impact of clinical factors on blood-based biomarkers for Alzheimer’s disease
Source: Transl Neurodegener. 2023 Aug 18;12:39. doi: 10.1186/s40035-023-00371-z (PMC10436434; doi:10.1186/s40035-023-00371-z)
Supplement: Supplementary file 2 — Additional file 2: Table S1. Clinical characteristics and plasma biomarkers of the study participants. Table S2. Comparisons of plasma biomarkers across age groups in different cognitive status. Fig. S1. Correlations between plasma biomarkers and age. Fig. S2. Correlations between plasma biomarkers and brain Aβ burden. [file 40035_2023_371_MOESM2_ESM.docx]

**Additional file 2.**

| Table S1. Clinical characteristics and plasma biomarkers of the study participants | | | | | | | | |
| --- | --- | --- | --- | --- | --- | --- | --- | --- |
| Index | | All (*n*=685) | CN (*n*=401) | | CI (*n*=284) | | | Z/χ^2^ (*P* value) |
| ***Demographics*** | | | | | | | | |
| Age(years) | 65 (60, 70) | | 65 (59, 69) | | 67 (60, 71) | | | -2.573 (0.010) |
| Gender [Male, *n* (%)] | 264 (38.5%) | | 152 (37.9%) | | 112 (39.4%) | | | 0.165 (0.685) |
| Education(years) | 12 (9, 14) | | 12 (10, 15) | | 10 (8, 12) | | | -8.101 (<0.001) |
| BMI (kg/m^2^) | 23.2 (21.3, 25.3) | | 23.5 (21.4, 25.9) | | 23.1 (21.2, 24.9) | | | -2.109 (0.035) |
| Decreased grip strength [*n* (%)] | 168 (24.5%) | | 74 (18.5%) | | 94 (33.1%) | | | 20.421 (<0.001) |
| Sleep duration (hours) | 6.5 (5.5, 7.5) | | 6.5 (6.0, 7.0) | | 7.0 (5.5, 8.0) | | | -2.238 (0.025) |
| APOE ε4+ [*n* (%)] | 178 (26.0%) | | 80 (20.0%) | | 98 (34.5%) | | | 18.317 (<0.001) |
| Current smoker [*n* (%)] | 54 (7.9%) | | 27 (6.7%) | | 27 (9.5%) | | | 1.762 (0.184) |
| Current drinker [*n* (%)] | 66 (9.6%) | | 37 (9.2%) | | 29 (10.2%) | | | 0.185 (0.667) |
| ***Neuropsychological tests*** | | | | | | | | |
| MMSE | 27 (25, 29) | | 28 (27, 29) | | 25 (19, 27) | | | -14.869 (<0.001) |
| MoCA-BC | 24 (20, 27) | | 26 (24, 27) | | 19 (14, 23) | | | -16.526 (<0.001) |
| ACE-III-CV | 76 (66, 84) | | 81 (75, 87) | | 64 (52, 73) | | | -15.895 (<0.001) |
| AVLT delayed recall | 4 (2, 6) | | 5 (3, 7) | | 2 (0, 3) | | | -13.970 (<0.001) |
| AVLT recognition | 21 (19, 23) | | 22 (21, 23) | | 18 (16, 20) | | | -15.734 (<0.001) |
| BNT (30 items) | 23 (20, 26) | | 25 (22, 27) | | 20 (18, 23) | | | -10.251 (<0.001) |
| AFT | 15 (12, 18) | | 16 (14, 20) | | 12 (9, 14) | | | -12.331 (<0.001) |
| STT-A | 47 (39, 58) | | 44 (37, 54) | | 53 (45, 72) | | | -7.729 (<0.001) |
| STT-B | 130(103, 161) | | 117(97, 146) | | 152(126, 193) | | | -9.153 (<0.001) |
| JLO | 21 (17, 24) | | 22 (18, 25) | | 19 (16, 22) | | | -6.376 (<0.001) |
| SDMT | 35 (27, 44) | | 40 (31, 46) | | 29 (19, 37) | | | -10.569 (<0.001) |
| DST | 12 (11, 14) | | 12 (11, 14) | | 12 (10, 13) | | | -3.764 (<0.001) |
| ADL | 20 (20, 20) | | 20 (20, 20) | | 20 (20, 21) | | | -8.851 (<0.001) |
| FAQ | 0 (0, 1) | | 0 (0, 0) | | 0 (0, 4) | | | -8.483 (<0.001) |
| HAMD | 3 (1, 7) | | 3 (0, 6) | | 4 (1, 8) | | | -2.534 (0.011) |
| HAMA | 4 (1, 8) | | 4 (1, 8) | | 4 (1, 8) | | | -0.249 (0.804) |
| ***Comorbidities*** | | | | | | | | |
| Fazekas scores | | | | | | | | |
| 0 [*n* (%)] | 364 (53.1%) | | 277 (56.6%) | | 137 (48.2%) | | | 28.294 (<0.001) |
| 1 [*n* (%)] | 177 (25.8%) | | 101 (25.2%) | | 76 (26.8%) | | |  |
| 2 or 3 [*n* (%)] | 68 (9.9%) | | 23 (5.7%) | | 45 (15.8%) | | |  |
| IHD [*n* (%)] | 47 (6.9%) | | 32 (8%) | | 15 (5.3%) | | | 1.894 (0.169) |
| Hypertension [*n* (%)] | 231 (33.7%) | | 136 (33.9%) | | 95 (33.5%) | | | 0.016 (0.899) |
| Hyperlipidemia [*n* (%)] | 117 (17.1%) | | 69 (17.2%) | | 48 (16.9%) | | | 0.011 (0.917) |
| Diabetes [*n* (%)] | 102 (14.9%) | | 64 (16%) | | 38 (13.4%) | | | 0.873 (0.350) |
| Cerebrovascular disease [*n* (%)] | 33 (4.8%) | | 14 (3.5%) | | 19 (6.7%) | | | 3.710 (0.054) |
| Hypothyroidism [*n* (%)] | 23 (3.4%) | | 18 (4.5%) | | 5 (1.8%) | | | 3.814(0.051) |
| Liver diseases [*n* (%)] | 26 (3.8%) | | 18 (4.5%) | | 8 (2.8%) | | | 1.273 (0.259) |
| CKD [*n* (%)] | 25 (3.6%) | | 21 (5.2%) | | 4 (1.4%) | | | 6.930 (0.008) |
| ***Plasma biomarkers*** | | | | | | | | |
| Aβ42 (pg/ml) | 9.96 (7.83, 12.01) | | | 10.06 (8.00, 12.03) | | 9.84 (7.60, 11.95) | -1.069 (0.285) | |
| Aβ40 (pg/ml) | 194.60 (161.89, 233.61) | | | 195.08(162.57, 228.46) | | 194.12 (161.71, 240.00) | -0.883 (0.377) | |
| Aβ42/Aβ40 ratio | 0.0515 (0.0433, 0.0605) | | | 0.0528 (0.0451, 0.0614) | | 0.0499 (0.0412, 0.0594) | -2.807 (0.005) | |
| T-tau (pg/ml) | 2.17 (1.69, 2.93) | | | 2.20 (1.72, 2.92) | | 2.16 (1.66, 2.99) | -0.315 (0.752) | |
| P-tau181 (pg/ml) | 1.87 (1.33, 2.83) | | | 1.71 (1.28, 2.47) | | 2.22 (1.48, 3.68) | -5.995 (<0.001) | |
| NfL (pg/ml) | 14.39(10.37, 19.96) | | | 12.67 (9.31, 17.69) | | 17.33(12.12, 22.41) | -7.374 (<0.001) | |
| ***AV45 PET SUVR*** | 1.23 (1.17, 1.31) | | | 1.22 (1.16, 1.28) | | 1.25 (1.17, 1.42) | -4.638 (<0.001) | |
| Data are show*n* as media*n* (interquartile range) or *n* (%). Group comparisons were performed using the Mann-Whitney U test or Chi-squared test based o*n* the data type.  Abbreviations: CN, cognitively normal; CI, cognitively impairment; BMI, body mass index; APOE, apolipoprotei*n* E; MMSE, Mini-Mental State Examination; MoCA-BC, Chinese versio*n* of Montreal Cognitive Assessment-Basic; ACE-III-CV, Chinese versio*n* of Addenbrooke’s Cognitive Examinatio*n* III; AVLT, Auditory Verbal Learning Test; BNT, Bosto*n* Naming Test; AFT, Animal Verbal Fluency Test; STT-A and B, Shape Trail Test Part A and B; ADL, Activities of Daily Living; JLO, Judgement of Line Orientation; SDMT, Symbol Digit Modalities Test; DST, Digit Spa*n* Test; Functional Assessment Questionnaire; HAMD, Hamilto*n* Depressio*n* Scale; HAMA, Hamilto*n* Anxiety Scale; IHD, Ischemic heart disease; CKD, Chronic kidney disease; AV45 PET, 18F-florbetapir positro*n* emissio*n* tomography; SUVR, standard uptake value ratios. | | | | | | | | |

| Table S2. Comparisons of plasma biomarkers across age groups in different cognitive status | | | | | | | | |
| --- | --- | --- | --- | --- | --- | --- | --- | --- |
| Age | <60 years | | 60∽70 years | | >70 years | | | Z (P value) |
| ***CN (n)*** | | 123 | | 202 | | 76 |  | |
| Aβ42/Aβ40 ratio | 0.0562(0.0482, 0.0646) | | 0.0518(0.0453, 0.0615)† | | 0.0502(0.0410, 0.0566)‡﹟ | | | 18.35(<0.001) |
| T-tau (pg/ml) | 2.11(1.72, 2.91) | | 2.21(1.80, 2.92) | | 2.27(1.62, 2.79) | | | 1.18(0.552) |
| P-tau181 (pg/ml) | 1.53(1.11, 2.01) | | 1.76(1.32, 2.49)† | | 2.12(1.47, 2.76)‡ | | | 17.97(<0.001) |
| NfL (pg/ml) | 9.42(7.50, 12.23) | | 13.37(10.13, 17.42)† | | 18.50(15.33, 22.95)‡﹟ | | | 100.75(<0.001) |
| ***CI (n)*** | | 74 | | 136 | | 73 |  | |
| Aβ42/Aβ40 ratio | 0.0515(0.0429, 0.0608) | | 0.0498(0.0410, 0.0587) | | 0.0475(0.0401, 0.0593) | | | 2.19(0.334) |
| T-tau (pg/ml) | 2.47(1.66, 3.24) | | 2.06(1.66, 2.71) | | 2.16(1.72, 3.03) | | | 3.72(0.155) |
| P-tau181 (pg/ml) | 2.15(1.51, 4.38) | | 1.98(1.42, 3.19) | | 2.57(1.83, 3.70) | | | 5.67(0.059) |
| NfL (pg/ml) | 14.16(10.03, 21.63) | | 16.02(11.98, 20.72) | | 20.67(16.45, 29.02)‡﹟ | | | 31.90(<0.001) |
| Data are shown as median (interquartile range). Group comparisons were performed using the Kruskal Wallis test.  Abbreviations: CN, cognitively normal; CI, cognitively impaired.  †P<0.05 pairwise comparison between the groups of age <60 years and age 60～70 years  ‡P<0.05 pairwise comparison between the groups of age <60 years and age >70 years  ﹟P<0.05 pairwise comparison between the groups of age 60～70 years and age >70 years | | | | | | | | |

Figure S1. Correlations between plasma biomarkers and age.

Abbreviations: CN, cognitively normal; CI, cognitively impaired.

Figure S2. Correlations between plasma biomarkers and brain Aβ burden.

Abbreviations: AV-45 PET SUVR, 18F-florbetapir PET standard uptake value ratios; CN, cognitively normal; CI, cognitively impaired.
